# Ultra-performance hydrophilic interaction liquid chromatography coupled with tandem mass spectrometry for simultaneous determination of allopurinol, oxypurinol and lesinurad in rat plasma: Application to pharmacokinetic study in rats

**DOI:** 10.1371/journal.pone.0213786

**Published:** 2019-03-14

**Authors:** Muzaffar Iqbal, Essam Ezzeldin, Rashed Naji Herqash, Ozair Alam

**Affiliations:** 1 Department of Pharmaceutical Chemistry, College of Pharmacy, King Saud University, Riyadh, Saudi Arabia; 2 Bioavailability Laboratory, College of Pharmacy, King Saud University, Riyadh, Saudi Arabia; 3 Medicinal Chemistry and Molecular Modelling Lab, Department of Pharmaceutical Chemistry, School of Pharmaceutical Education and Research, Jamia Hamdard, New Delhi, India; Indian Institute of Chemical Technology, INDIA

## Abstract

A fixed dose combination of lesinurad and allopurinol has been recently approved by USFDA and EMA for treatment of gout-associated hyperuricemia in patients who have not achieved target serum uric acid levels with allopurinol alone. In this study, an ultra-performance hydrophilic interaction liquid chromatography (UPHILIC) coupled with tandem mass spectrometry method was developed and validated for simultaneous determination of allopurinol, oxypurinol and lesinurad in rat plasma. Liquid liquid extraction using ethyl acetate as extracting agent was used for samples extraction procedure. Acquity UPLC HILIC column (100 mm x 2.1, 1.7μm) was used for separation of allopurinol, oxypurinol, lesinurad and internal standard (5-Florouracil). The mobile phase consisting of acetonitrile, water and formic acid (95:5:0.1, v/v/v), were eluted at 0.3 mL/min flow rate having total chromatographic run time of 3 min per sample. The analytes were detected on Acquity triple quadrupole mass spectrometer equipped with a Z-Spray electrospray ionization (ESI). The ESI source was operated in negative mode and multiple reaction monitoring was used for ion transition for all compounds. The precursor to product ion transition of m/z 134.94 > 64.07 for allopurinol, 150.89 > 41.91 for oxypurinol, 401.90 > 176.79 for lesinurad and 128.85 >41.92 for internal standard were used for identification and quantification. The calibration curves for all analytes were found to be linear with weighing factor of 1/x^2^ using regression analysis. The developed assay was successfully applied in an oral pharmacokinetic study of allopurinol, oxypurinol and lesinurad in rats.

## Introduction

Gout is a form of inflammatory arthritis characterized by the deposition of monosodium urate crystals in the joints due to elevated levels of serum uric acid (SUA), also known as hyperuricaemia [1,2]. Allopurinol (ALP), a xanthine oxidase inhibitor (XOI), is one of the most commonly prescribed medicine for the treatment of hyperuricaemia and gout [3,4]. It acts by inhibiting the xanthine oxidase (XO) enzyme which catalyzes the formation of xanthine from hypoxanthine and further to uric acid [3,5]. ALP is rapidly absorbed orally and subsequently metabolized by XO to a major active metabolite, oxypurinol (OXP). Like ALP, OXP also inhibits XO enzyme and has much longer serum half-life (⁓23 h) compared to ALP (⁓1.2 h) and therefore responsible for most of the pharmacological effects of ALP [6–8]. Although the main therapeutic effect produces by OXP, but due to poor absorption of OXP preparation, parent drug (ALP) is still used as main formulation [9].

In spite of recommended as first-line therapy, >50% of patients do not achieve sustained reductions in SUA levels (< 6 mg/dL) by the most commonly prescribe ALP dose of 300|mg/day [10–12]. Lesinurad (LES) is a novel and selective uric acid transporter 1 (URAT1) inhibitor, which lowers SUA levels via increasing renal uric acid excretion. It produce beneficial effects for the treatment of gout along with XOIs. Therefore, USFDA and EMA has approved a fixed-dose combination (FDC) of LES and ALP for once-daily treatment of gout-associated hyperuricemia in patients who have not achieved target SUA levels with ALP alone [13,14]. LES inhibits URAT1, a uric acid transporter responsible for the reabsorption of uric acid from the renal tubular lumen and therefore in combination with ALP provides a dual mechanism for SUA lowering: an increase in excretion of uric acid and reduction in urate production [15–17].

Due to lack of adherence to therapy and high inter-subject variability in OXP pharmacokinetics, therapeutic drug monitoring (TDM) during ALP therapy is usually recommended to establish the relationship between dose versus plasma concentration, renal function and SUA levels and to determine the minimum plasma concentration of OXP require to achieve the target SUA level of < 6mg/dL. [18,19]. Several methods have been reported in literature for simultaneous determination of ALP and OXP in human plasma [20–24]. Recently, Zhou XY et al, described the assay for the determination of LES in rat plasma by UPLCMS/MS method [25]. Since LES is therapeutically used in combination only with XOIs, and after approval of FDC of ALP and LES, a validated assay is required for simultaneous determination of ALP, OXP and LES in plasma. Herein, an ultra-performance hydrophilic interaction liquid chromatography interfaced with the electrospray ionization (ESI) source of a tandem mass spectrometer (UPHILIC-MS/MS) was used for development and validation of a novel assay for simultaneous determination of ALP, OXP and LES in rat plasma. The developed assay was successfully applied in an oral pharmacokinetic studies in rats.

## Materials and methods

**Fig 1:**
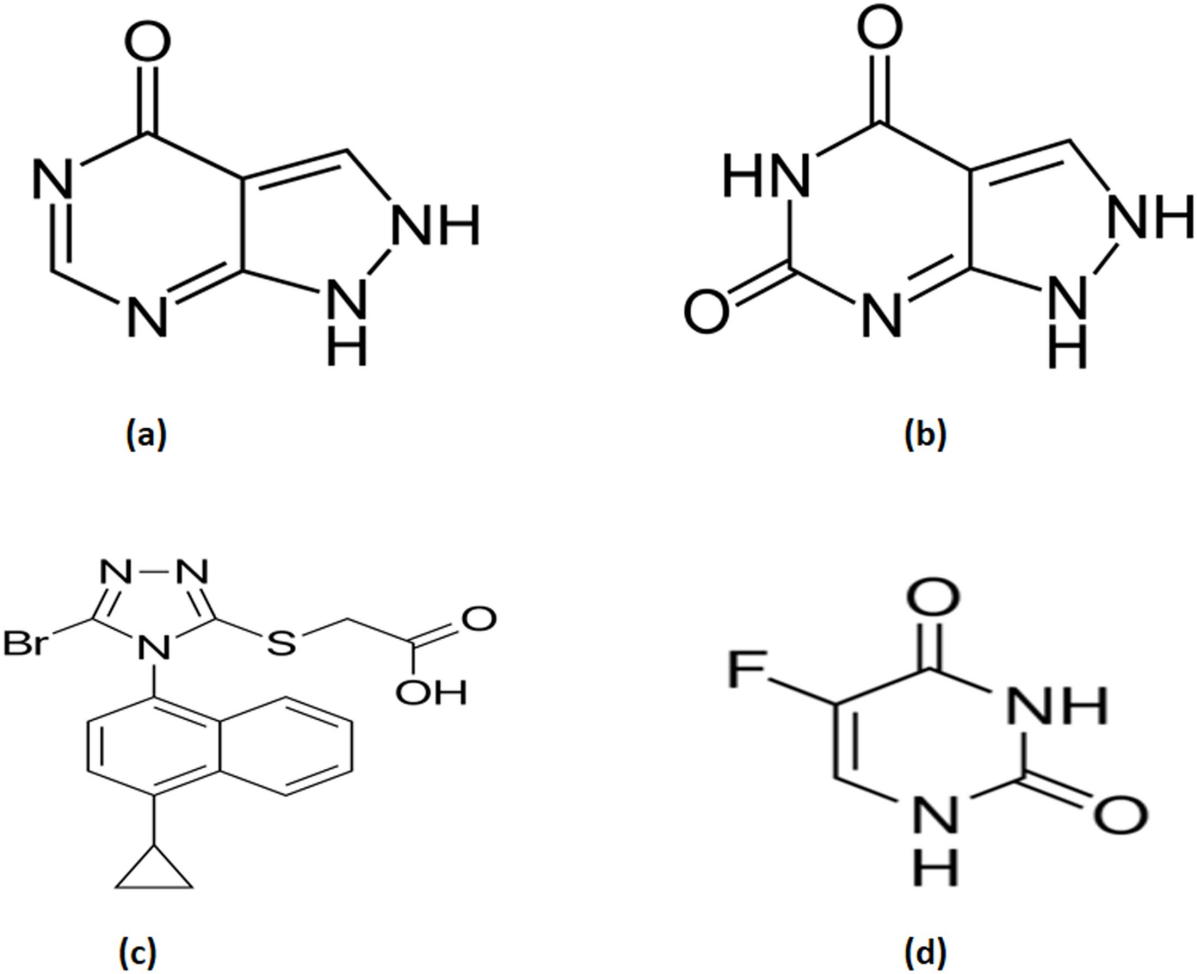
Chemical structure of (a) ALP (b) OXP (c) LES and (d) IS.

The standards of LES, OXP were purchased from Toronto Research Chemicals (North York, Ontario, Canada) while ALP and 5-florouracil (5-FU) were obtained from Qingyang Pharmaceutical Co., Ltd (Chongqing, China) and Alfa Aesar (Ward Hill, MA), respectively (Fig 1). HPLC grade methanol and acetonitrile were purchased from Sigma-Aldrich (St. Louis, MO, USA). Ethyl acetate, formic acid and sodium hydroxide were procured from Qualikem Fine Chemicals (Vadora, India). Dimethyl sulphoxide was purchased from Panreac Quimica (Barcelona, Spain). Milli-Q grade of deionized water (Millipore, Moscheim, Cedex, France) was used for aqueous solution preparation. Healthy wistar albino rats were used for blood harvesting for blank plasma and same strain was used for pharmacokinetic study. Experimental rats were obtained from “Animal Care and Use Centre, College of Pharmacy, King Saud University, Riyadh, Saudi Arabia” and were housed in standard condition. Animal utilization experiments were performed according to the “guidelines of the animal care and use committee at King Saud University” and approved protocol by the “Animal Ethics Committee, College of Pharmacy, King Saud University.”

### Preparation of stock, calibration standards (CSs) and quality controls (QCs) sample

Stock solution of ALP (0.4 mg/mL), OXP (0.4 mg/mL) and LES (1 mg/mL) were prepared by dissolving of their requisite amounts in methanol:1% NaOH in water (90:10, v/v), methanol and dimethyl sulphoxide, respectively. These stock solutions were further serially diluted with acetonitrile:water (70:30, v/v) to prepare the different range of working solutions. An appropriate amount of working solution were spiked in drug free plasma to achieve plasma CSs of ALP (22–8000 ng/mL), OXP (33–12000 ng/mL) and LES (25–9000 ng/mL) concentration, respectively. Same procedure was followed to prepare the low, medium and high QCs (LQC, MQC and HQC) of ALP (70, 1400 and 7000 ng/mL), OXP (100, 2000 and 10000 ng/mL) and LES (80, 1600 and 8000 ng/mL), respectively. The stock solution of 5-FU (0.5 mg/mL), which was used as internal standard (IS) was also prepared in methanol and was diluted with acetonitrile:water (70:30, v/v) to get working solution of 2 μg/mL. Both stock and working solutions were kept in pharmaceutical refrigerator maintained at 4°C ±2°C, while spiked plasma CCs and QCs samples were kept at -70 ±5°C until use.

### Chromatography and mass spectrometry condition

An Acquity UPLC system (Waters Corp, Miliford, MA, USA) consisting of quaternary solvent manager, pump, column oven and an autosampler was used for mobile phase delivery. Chromatographic separation was achieved on Acquity UPLC HILIC column (100 mm x 2.1, 1.7μm) maintained at 40°C ±5°C. The mobile phase consisting of acetonitrile, water and formic acid (95:5:0.1, v/v/v) and were eluted at 0.3 mL/min. The total chromatographic run time was 3 min per sample.

The analytes were detected on Acquity triple quadrupole (TQD) mass spectrometer (Waters Corp, Miliford, MA, USA) equipped with a Z-Spray electrospray ionization (ESI). The ESI source was operated in negative mode and multiple reaction monitoring (MRM) was used for precursor to product ion transition for all compounds. The optimized source operation conditions were as follows: capillary voltage, 2.80 kV; extractor voltage, 3.0 kV; source temperature, 150 °C; desolvation temperature, 350 °C; desolvation gas flow rate, 400 L/h; collision gas flow rate, 0.17 mL/min. Highly pure nitrogen was used as collision gases while argon was used as collision energy flow. The Mass Lynx software (Version 4.1) was used to operate the UPLC-MS/MS system and all the collected data were processed by the Target Lynx program.

### Sample preparation

All plasma samples were initially thawed, vortex-mixed and allowed to equilibrate at room temperature before processing. An aliquot of 0.2 mL of plasma samples, 10 μL of IS (2 μg/mL) was added excluding blank sample. The samples were vortex-mixed and 2 mL of ethyl acetate was added into each samples. Then samples were vortex-mixed gently for one min followed by shaking for 10 min on shaker. The samples were transferred for cold centrifugation (4 °C) at 4000 rpm. After centrifugation, the separated organic layer was transferred into fresh tube and were dried in sample concentrator maintained at medium temperature (Thermo Scientific N.C. USA). The dried sample was reconstituted by using 200 μL of acetonitrile, vortex-mixed and 5 μL was injected in UPLC-MS/MS for analysis.

### Method validation

The method was validated according to guideline for bioanalytical method validation specified by USFDA [26]. Assay linearity, selectivity and specificity, accuracy and precision, recovery and matrix effects and stability parameters were evaluated.

#### Selectivity and specificity

Selectivity and specificity for the assay was evaluated to ensure the absence of interferences from endogenous components of plasma. It was investigated by comparing the responses of the blank plasma chromatograms (obtained from six different healthy rats) with chromatogram of plasma spiked with lower limit of quantification (LLOQ) concentration for each analyte.

#### Assay linearity and LLOQ

The quantification of ALP, OXP and LES has been carried out by using IS method. The plasma calibration curves (CCs) of each analyte was derived by plotting the graph between peak area ratios of analyte to IS versus the nominal concentration of analyte. The assay linearity of CCs was derived by back calculation of concentration for each analyte using least square regression model with weighing factor of 1/X^2^. The LLOQ was defined as the lowest concentration of the CCs having signal-to noise ratio (S/N) ratio of 10:1 and can be quantified with accuracy and precision within ±20%.

#### Accuracy and precision

Precision and accuracy of ALP, OXP and LES were determined at LLOQ, LQC, MQC and HQC concentrations in spiked plasma samples in five replicates. Both intra-day (single day) and interday (three consecutive day) precision and accuracy were measured. The relative standard deviation (RSD, %) was used to express the precision while percentage difference in measured concentration with nominal concentration was used to express the accuracy of the assay.

#### Extraction recovery and matrix effects

The extraction recovery and matrix effects were evaluated at LQC, MQC and HQC concentration levels for all analytes (ALP, OXP and LES) and at 100 ng/mL concentration for IS. The percentage recovery was calculated by comparing the measured peak response of analytes spiked before extraction (pre-extraction) with those spiked after the extraction (post extraction). The matrix effect which expressed as ion suppression/ion enhancement effects were determined by comparing the responses of plasma spiked with analytes after extraction (post extraction) with those of aqueous solution.

#### Stability

The stability of ALP, OXP and LES in spiked plasma under different anticipated storage conditions were evaluated at their LQC and HQC concentration levels in five replicates. The short-term (at room temperature for 8 h); post preparation (in autosampler at 10 °C for 24 h), freeze-thaw (three cycle from ─80 °C to room temperature) and long-term (─80 °C for 30 days) stabilities were determined. All stabilities results were measured against freshly spiked CCs and acceptance were limited to be within ±15%.

### Pharmacokinetic studies in rats

Six healthy rats weighing between 180–220 g were received and maintained under standard laboratory conditions before the experiments. An accurately weighed amount of ALP and LES were mixed with carboxyl methyl cellulose (0.5%, w/v) to prepare an oral formulation in form of suspension. Animals were fasted for 12 h prior to the administration of oral doses of 15 mg/kg of LES and 20 mg/kg for ALP. Blood samples (approx.0.5 ml) were collected at 0, 0.33, 0.66, 1, 2, 3, 5, 8, 12 and 24 h in lithium heparinized tubes after dosing. Plasma was separated by centrifugation for 8 min at 4000 rpm and stored at −80 °C till further analysis.

The non-compartmental model was used to calculate the pharmacokinetic parameters using WinNonlin software (Pharsight Co., Mountain View, CA, USA). The maximum plasma concentration (C_max_), time to achieve this (T_max_), area under curve zero to last and infinity (AUC_0-24_; AUC_0-∞_), half-life (T_½_), elimination rate constant (Kel) and mean residence time were measured. Statistical parameters like mean, standard deviation and % RSD were calculated by using MS-Excel 2013 (Microsoft).

## Results and discussion

### Optimization of mass spectrometry conditions

**Fig 2:**
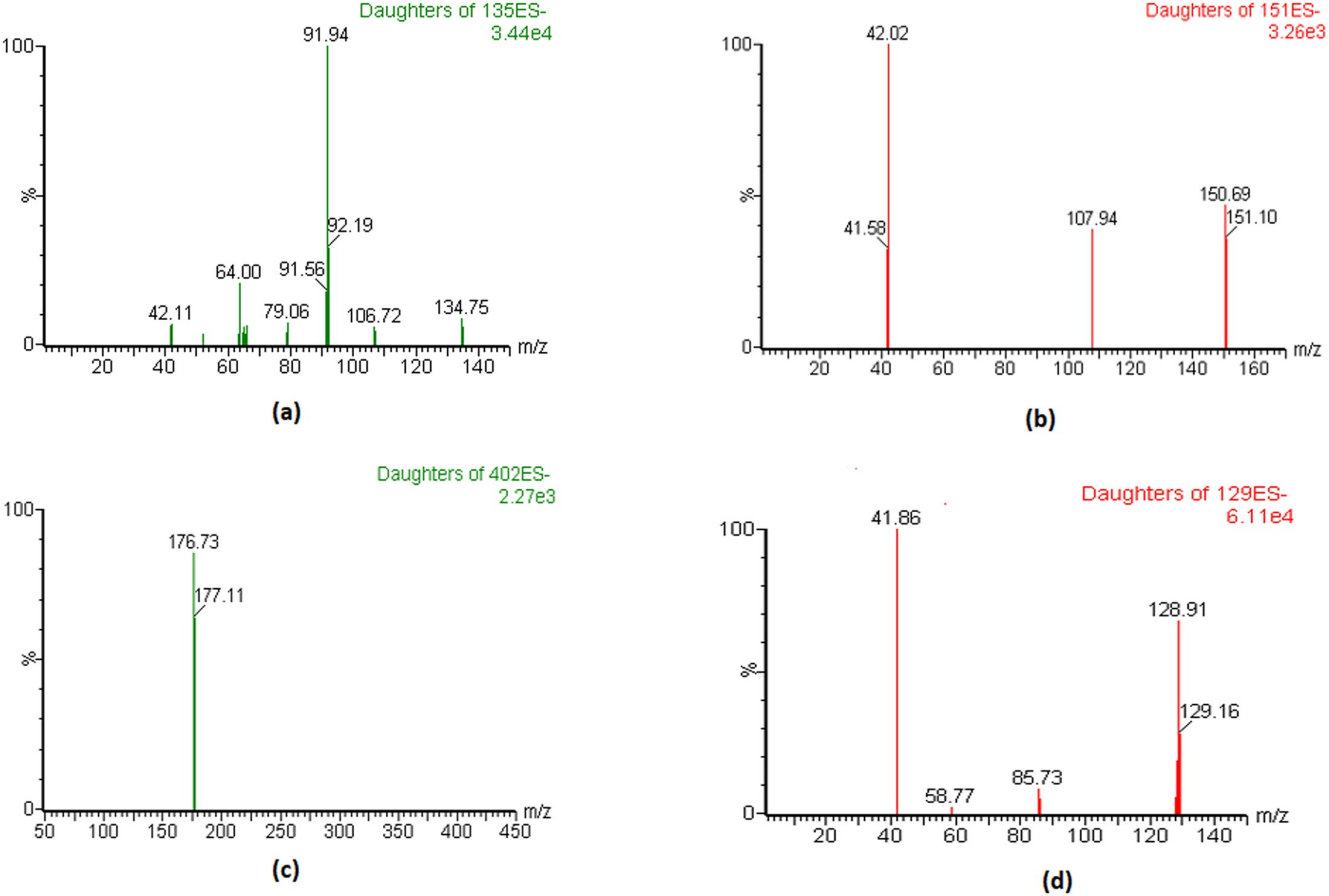
Fragment ions spectra of (a) ALP (b) OXP (c) LES and (d) IS.

In previous literatures, ALP and OXP were detected by both ESI positive or negative mode, whereas LES was only measured by ESI positive mode. For optimization of ionization process, tuning experiment was performed by direct injection of 500 ng/mL of respective standard solutions into mass spectrometer under positive and negative ESI mode. Herein, like previous studies, ALP and OXP were found to be sensitive in both positive as well as negative mode, however the deprotonated precursor ions produced in negative mode (m/z 134.94 for ALP and 150.89 for OXP) was more stable than the protonated precursor ion in positive mode. Although LES has shown better signal response in positive mode, but was also found to produce stable deprotonated precursor ions at m/z 401.90 in negative mode. Thus negative ionization mode was selected for monitoring of all three analytes (ALP, OPX and LES) which helped in curtailing time required for stabilization of high voltages during polarity switching. The IS, 5-FU was also found to be sensitive in negative mode which produced deprotonated precursor ions at m/z of 128.85 and was selected for IS for this study. The selected specific transition and optimized compound specific parameters are listed in Table 1, whereas the spectral representation of fragment ion transition for ALP, OXP, LES and IS are shown in Fig 2.

**Table 1:** Optimized compound specific parameters for ALP, OXP, LES and IS.

Compound	Q1 [M-H]ˉ	CV (V)	Q3 [M-H]ˉ	CE (eV)	*dt* (sec)
ALP	134.94	32	64.07	20	0.077
OXP	150.89	40	41.91	16	0.077
LES	401.90	22	176.79	17	0.077
IS (5-FU)	128.85	32	41.92	14	0.077

Q1 = parent ion; CV = cone voltage; dt = dwell time; Q3 = fragment ion [M-H]ˉ CE = collision energy)

### Optimization of chromatographic conditions

The optimization of chromatographic procedure was aimed to separate all three target compound (ALP, OPX and LES) and IS (5-FU) with high resolution and good peak shape. Initially, the reverse phase chromatography was tried by using Acquity UPLC BEH C_18_, 1.7μm column, but no simultaneous separation of all compounds together was achieved. Then HILIC method was tried due to its property of normal phase and reversed phase as well as the ion chromatography. By using Acquity UPHILIC column, 1.7μm, we were able to separate all compounds together. Previously, OXP and 5-FU has been already separated by HILIC method [27–28]. For mobile phase composition optimization, we injected the mixed solution of all compounds to the system and their responses were monitored under different composition. The peak shape of LES was better once the mixture was eluted with methanol as organic modifiers, but the response of ALP and OXP was not appropriate. Therefore, the mobile phase using ACN as organic modifier with different content of ammonium acetate/formate and formic acid were tried. Formic acid was selected as volatile buffer because, no adequate separation of LES was observed with ammonium acetate/formate. The different mobile phase composition comprising of acetonitrile and 0.1% formic acid in water were tried in ratio of 97/3; 95/5 and 90/10. Finally, the mobile phase consisting of acetonitrile, water and formic acid (95:5:0.1, v/v/v) were eluted at 0.3 mL/min and was fixed for separation of all target compounds.

### Method validation

#### Selectivity and specificity

**Fig 3:**
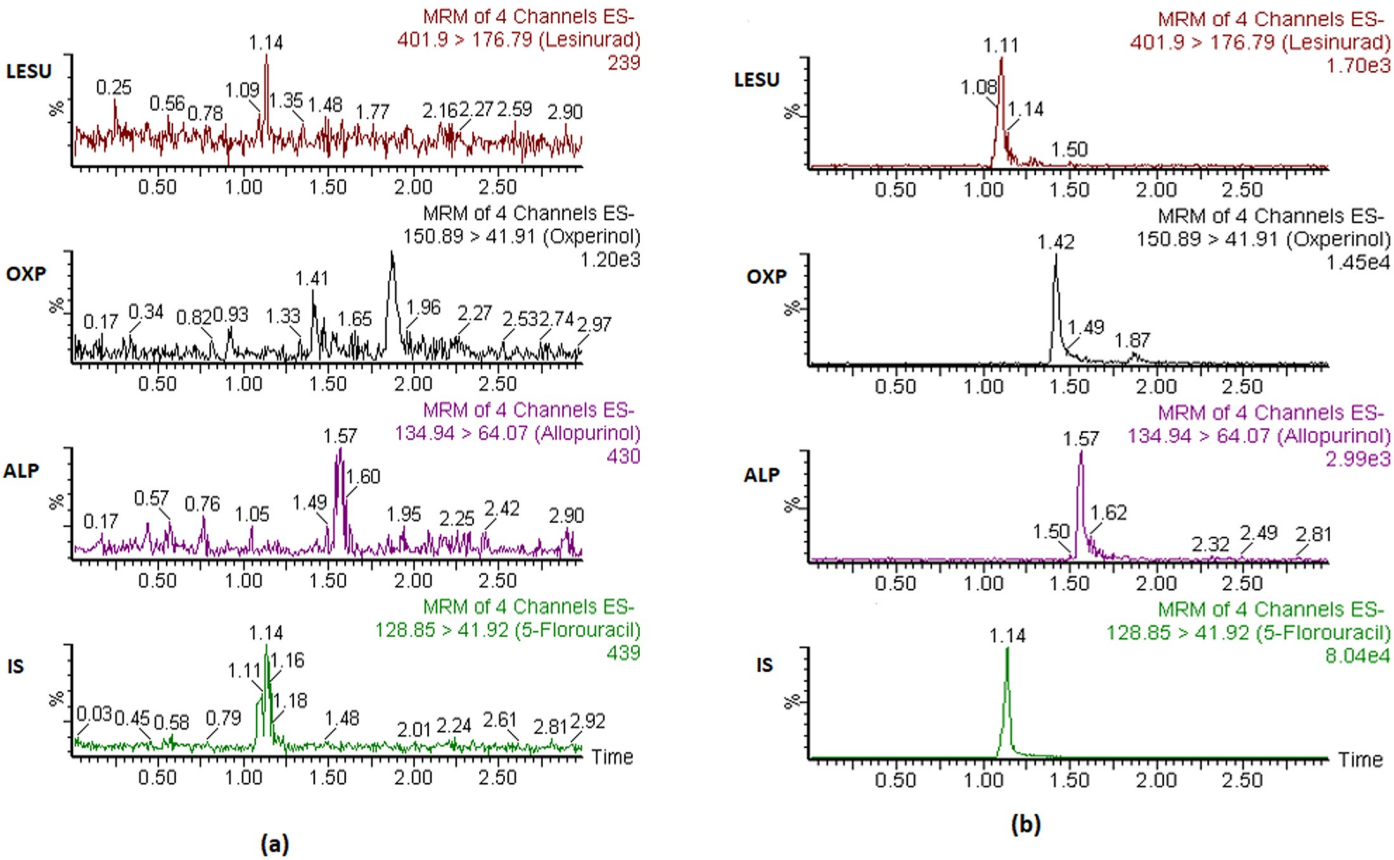
The representative MRM chromatograms of ALP, OXP, LES and IS in (a) blank rat plasma and (b) plasma spiked at LLOQ concentrations.

There were no significant interferences observed in the MRM chromatograms of ALP, OXP and LES of the blank plasma samples at their respective retention times in comparison with that of plasma spiked with LLOQ standards. This results illustrate that the developed assay is highly selective and specific for the detection of all these three analytes in real plasma samples. The representative MRM chromatograms of ALP, OXP, LES and IS in blank plasma and plasma spiked at LLOQ level are shown in Fig 3.

#### Assay linearity and LLOQ

The CCs were found to be linear between 22–8000 ng/mL for ALP; 33–12000 ng/mL for OXP and 25–9000 ng/mL for LES, respectively which was acceptable to cover the therapeutic reference ranges and expected plasma concentrations in the pharmacokinetic study. The coefficient of determination (r^2^) value was found to be within the range of 0.991–0.999 for all analytes by using regression analysis of weighing factor of 1/x^2^ fitted for each CCs. The mean back calculated concentrations of each CSs (except lowest concentration) point were found to be within the acceptable limits of precision (± 15%) and accuracy (≤ 15%) for all analyte. While the lowest concentration of the CSs (22 ng/mL for ALP; 33 ng/mL for OXP and 25 ng/mL for LES) was quantified with acceptable accuracy (± 20%) and precision (≤ 20%, % CV) and therefore was defined as LLOQ of the assay.

#### Accuracy and precision

Table 2 represents a summarize view of accuracy and precision data for all three analytes at their four different respective QC concentration levels (LLOQ, LQC, MQC and HQC). The intra and inter-day precision (% RSD) for ALP, OXP and LES were found to be ≤10.54, ≤13.98 and ≤14.84%, respectively, whereas the intra and interday accuracy were within range of 90.40–111.21%, 95.16–111.13% and 91.92–108.30%, respectively. These results confirm that the developed assay was accurate and precise for all three analytes and therefore suitable for routine pharmacokinetic analysis.

**Table 2:** Inter and inter-day precision and accuracy data of ALP, OXP and LES in rat plasma.

Nominal QC (ng/mL)	Precision (RSD, %)		Accuracy (%)	
	Intra-day(n = 5)	Inter-day (n = 15)	Intra-day (n = 5)	Inter-day (n = 15)
**ALP**				
22	9.53	10.54	110.55	111.21
70	5.68	6.73	90.45	94.90
1400	3.43	5.69	96.40	92.58
7000	2.93	5.62	93.74	91.08
**OXP**				
33	13.84	13.98	111.13	111.01
100	5.96	7.00	108.71	102.10
2000	10.76	9.12	99.78	95.16
10000	1.90	7.18	105.04	99.29
**LES**				
25	11.00	9.99	98.49	108.30
80	14.84	9.82	92.89	91.92
1600	3.08	3.08	99.08	96.97
8000	7.49	10.12	104.68	94.67

#### Recovery and matrix effects

Table 3 represent the extraction recovery and matrix factor data of ALP, OXP, LES and IS measured at their respective, LQC, MQC and HQC level concentrations. The mean absolute recovery of ALP, OXP and LES were found to be 79.42, 66.89 and 55.95% respectively. Although the % recovery of LES was little bit lower compared to ALP and OXP, but was consistent and concentration independent between QCs concentration. The matrix effects represented as % matrix factor (% MF) was measured by post extraction (quantitative) method. The mean absolute % MF of ALP, OXP and LES were found to be 96.15, 91.35 and 94.55% respectively. This results confirm that slight ion suppression effects were observed as variability due to matrix effects and were ≤15% for all analytes which is within the acceptable limits and therefore not significant enough to influence analyte ionization.

**Table 3:** Extraction recovery and matrix effects data of ALP, OXP, LES and IS in rat plasma (n = 5).

Compound	QC level	ER %		MF %	
		% Mean ± SD	% RSD	%Mean ± SD	% RSD
**ALP**	LQC	76.88±4.92	6.39	100.55±2.19	2.17
	MQC	78.64±9.28	11.80	95.80±7.79	8.13
	HQC	82.73±5.95	7.20	92.10±3.23	3.50
	Mean	79.42±2.99	3.77	96.15±4.23	4.40
**OXP**	LQC	62.52±5.73	9.16	93.38±1.49	2.08
	MQC	71.05±3.33	4.69	91.23±3.57	3.92
	HQC	67.12±9.31	13.87	89.43 ± 3.79	3.33
	Mean	66.89±4.27	6.38	91.35±1.97	2.16
**LES**	LQC	63.05±3.73	5.91	95.13±5.79	5.99
	MQC	53.47±3.80	7.13	98.32±6.47	6.58
	HQC	51.33±4.24	8.26	90.19±6.82	7.56
	Mean	55.95±6.24	11.20	94.55±4.09	4.30
**IS**	100 ng/mL	72.23±7.51	10.40	88.47±3.70	4.20

#### Stability

The stability of all three analytes (ALP, OXP and LES) in spiked rat plasma samples which were tested at their respective LQC and HQC concentrations were found to be acceptable limits for both short term (room temperature) and long term storage (‒ 80 °C for 30 days). In addition, the process plasma samples of all spiked analytes were also found to be stable in autosampler for 24 h and after exposing them for three freeze-thaw cycles which provide additional confidence to the accuracy of the assay.

### Application to pharmacokinetic study in rats

**Fig 4:**
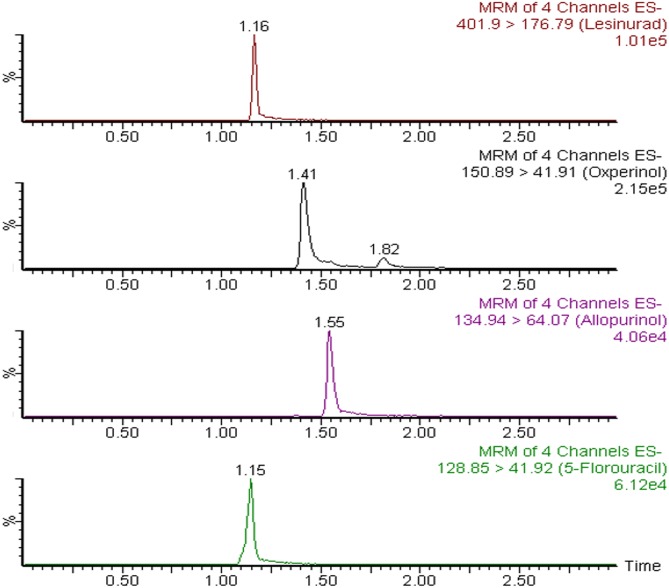
The representative MRM chromatograms of ALP, OXP, LES and IS at one h after oral administration of ALP (20 mg/kg) and LES (15 mg/kg).

**Fig 5:**
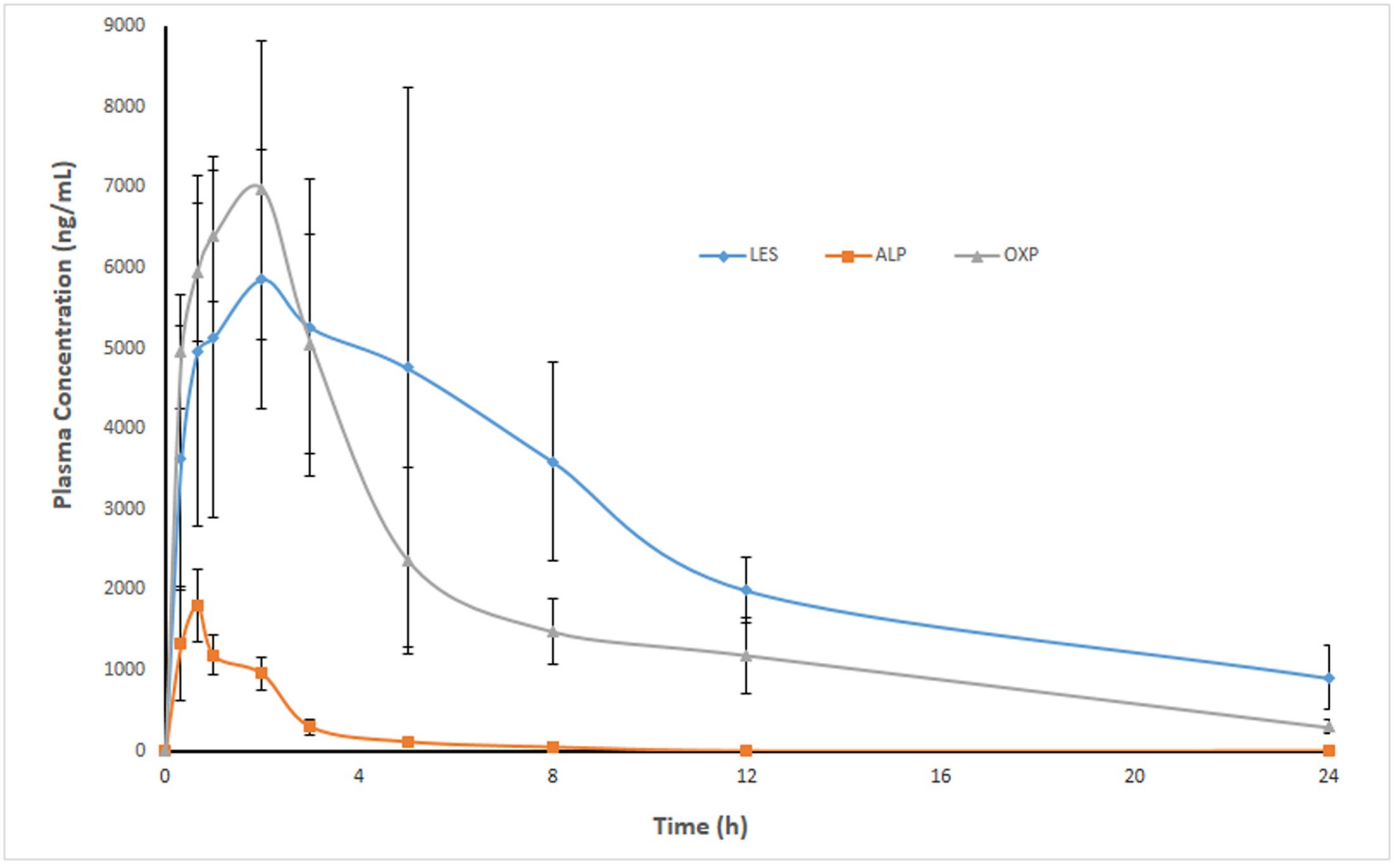
The plasma concentration-time profile of ALP, OXP, LES after oral administration of ALP (20 mg/kg) and LES (15 mg/kg).

The pharmacokinetic parameters result for ALP, OXP and ALP are summarized in Table 4. The results showed that ALP is extensively metabolized to OXP as it was undetectable after 8 h after post administration of 20 mg/kg of ALP. The T_max_ and T_1/2_ of ALP was 0.66 and 1.39 min only as compared to 2 and 6.11 min of OXP. Similarly, the rate (C_max_) and extent (AUC) of absorption of OXP was 3.7 and 12.7 times higher than ALP which confirms that the OXP is responsible for most of the pharmacological effects of ALP. LES is also rapidly absorbed with C_max_ of 8271 ng/mL, which was achieved at 1.5 h after 15 mg/kg of post oral administration. The ratios of AUC (AUC_0-24_/ AUC_0-∞_) were found to be ≥85% for ALP, OXP and LES, demonstrating that our assay was sensitive enough to cover the elimination phase of these analytes. The representative MRM chromatogram of ALP, OXP, LES and IS after 1 h of oral administration of ALP (20 mg/kg) and LES (15 mg/kg) are shown in Fig 4 whereas the mean plasma concentration-time curves of ALP, OXP and LES in rats are shown in Fig 5.

**Table 4:** The non-compartmental pharmacokinetic parameters for ALP, OXP and LES in rat plasma after oral administration of mg/kg of ALP and 15 mg/kg of LES.

Pharmacokinetic Parameters	Unit	ALP	OXP	LES
		Values (mean ± SD)		
**C**_**max**_	ng/mL	2028 ± 509	7495±156	8271 ± 191
**T**_**max**_	h	0.66	2	1.5
**AUC**_**0-24**_	ng.h/mL	3511 ± 571	44800±373	65909±139
**AUC**_**0-inf**_	ng.h/mL	3659±619	47408± 354	77144±147
**T**_**1/2**_	h	1.39±0.44	6.11±1.61	8.49±3.61
**MRT**	h	1.98±0.32	7.58±1.52	12.45±4.51
**Kel**	h^-1^	0.53±0.16	0.12±0.02	0.09±0.03

## Conclusions

An efficient and selective UPHILIC-MS/MS assay was developed and validated for simultaneous determination of ALP, OXP and ALP in rat plasma sample. UPHILIC based elution procedure coupled with tandem mass spectrometry enhanced the fast separation for simultaneous analysis of all three target compound within three min. This method was shown good linearity with high sensitivity, selectivity and precision as per regulated guideline. Sample extraction by liquid liquid extraction procedure using ethyl acetate produced negligible matrix effects and satisfactory recovery for all three target compound. The developed assay was successfully applied in pharmacokinetic characterization of all three compound in rats. Therefore, the assay can be used for pharmacokinetic study and TDM of ALP, and LES once they co-administered.

## Supporting information

S1 File The calibration curve of LES (a), ALP (b) and OXP (c) in spiked plasma samples (Figure A). Stability data under different storage conditions (Table A). The individual animal pharmacokinetic data of ALP (Table B), OXP (Table C), and LES (Table D).
(ZIP)
