# Supplementary material for: Ultra-performance hydrophilic interaction liquid chromatography coupled with tandem mass spectrometry for simultaneous determination of allopurinol, oxypurinol and lesinurad in rat plasma: Application to pharmacokinetic study in rats
Source: PLoS One. 2019 Mar 14;14(3):e0213786. doi: 10.1371/journal.pone.0213786 (PMC6417734; doi:10.1371/journal.pone.0213786)
Supplement: S1 File — Stability data under different storage conditions (Table A). The individual animal pharmacokinetic data of ALP (Table B), OXP (Table C), and LES (Table D). (ZIP) [file pone.0213786.s001.zip › Table A-D.docx]

| **Table A.** **Stability data of ALP, OXP and LES in rat plasma matrix under different storage conditions** | | | | | | | | | |
| --- | --- | --- | --- | --- | --- | --- | --- | --- | --- |
| Analytes | Nominal value (ng/mL) | Freeze/Thaw (3 cycle) | | Short term (8 h) | | long term (1 month) | | Processed samples (24 h) | |
|  |  | Precision (RSD, %) | Accuracy (%) | Precision (RSD, %) | Accuracy  (%) | Precision (RSD, %) | Accuracy  (%) | Precision (RSD, %) | Accuracy (%) |
| ALP | 70 | 12.72 | 108.60 | 3.97 | 93.08 | 3.57 | 87.23 | 8.54 | 96.54 |
|  | 7000 | 4.89 | 100.54 | 2.31 | 90.84 | 8.31 | 88.71 | 7.10 | 99.34 |
| OXP | 100 | 3.31 | 102.55 | 9.99 | 96.85 | 7.84 | 92.43 | 3.45 | 104.72 |
|  | 10000 | 7.02 | 92.67 | 3.46 | 95.12 | 5.34 | 91.04 | 4.56 | 102.65 |
| LES | 80 | 2.38 | 94.8 | 6.37 | 91.26 | 8.20 | 89.81 | 4.82 | 112.54 |
|  | 8000 | 4.97 | 90.13 | 3.46 | 95.12 | 5.01 | 88.59 | 2.56 | 97.01 |

| **Table B: Individual animal pharmacokinetic parameters data of ALP** | | | | | | | |
| --- | --- | --- | --- | --- | --- | --- | --- |
| Rats | Cmax | Tmax | AUClast | AUCtot | Kel | thalf | MRT |
| R1 | 1376.57 | 0.33 | 3315.05 | 3388.2 | 0.482538 | 1.43646 | 2.04163 |
| R2 | 1667.45 | 0.66 | 2635.21 | 2743.88 | 0.679192 | 1.02055 | 1.60682 |
| R3 | 2378.66 | 0.66 | 3641.95 | 3774.36 | 0.346061 | 2.00296 | 2.23271 |
| R4 | 2694.26 | 0.33 | 4404.97 | 4653.35 | 0.373019 | 1.85821 | 2.47707 |
| R5 | 2321.62 | 0.66 | 3470.35 | 3656.48 | 0.629286 | 1.10148 | 1.77685 |
| R6 | 1731.2 | 0.66 | 3598.67 | 3742.26 | 0.724413 | 0.95684 | 1.80062 |
| Mean | 2028.293 | 0.66 | 3511.033 | 3659.755 | 0.539085 | 1.396083 | 1.989283 |
| SD | 509.0235 |  | 571.152 | 619.6374 | 0.161336 | 0.448161 | 0.324633 |
| CV % | 25.09615 |  | 16.26735 | 16.93112 | 29.92774 | 32.10131 | 16.31909 |

| **Table C: Individual animal pharmacokinetic parameters data of OXP** | | | | | | | |
| --- | --- | --- | --- | --- | --- | --- | --- |
| Rats | Cmax | Tmax | AUClast | AUCtot | Kel | thalf | MRT |
| R1 | 6252.94 | 2 | 39476.6 | 41925.3 | 0.112351 | 6.16946 | 8.33495 |
| R2 | 7192.39 | 2 | 49418.6 | 50759.5 | 0.151146 | 4.58593 | 6.95621 |
| R3 | 10535.3 | 2 | 48647.7 | 50350.9 | 0.132908 | 5.21524 | 5.83747 |
| R4 | 7573.56 | 3 | 44044.7 | 45393.1 | 0.136171 | 5.09026 | 6.05867 |
| R5 | 6585.93 | 0.66 | 42639.7 | 45993.3 | 0.105613 | 6.56311 | 8.73074 |
| R6 | 6831.19 | 1 | 44576.2 | 50026.1 | 0.076616 | 9.04698 | 9.5786 |
| Mean | 7495.218 | 2 | 44800.58 | 47408.03 | 0.119134 | 6.11183 | 7.582773 |
| SD | 1558.906 |  | 3735.299 | 3545.951 | 0.026613 | 1.612323 | 1.525023 |
| CV % | 20.79867 |  | 8.337613 | 7.479643 | 22.33887 | 26.38037 | 20.11168 |

| **Table D: Individual animal pharmacokinetic parameters data of LES** | | | | | | | |
| --- | --- | --- | --- | --- | --- | --- | --- |
| Rats | Cmax | Tmax | AUClast | AUCtot | Kel | thalf | MRT |
| R1 | 5494.01 | 1 | 48246 | 69847.6 | 0.051008 | 13.589 | 20.1145 |
| R2 | 8320.88 | 3 | 64836.4 | 68738 | 0.124355 | 5.57396 | 8.70855 |
| R3 | 6878.96 | 2 | 61847.4 | 73776.1 | 0.076048 | 9.1146 | 12.6844 |
| R4 | 11031.4 | 5 | 85561.6 | 90181.7 | 0.135214 | 5.12629 | 8.47081 |
| R5 | 8808.81 | 0.66 | 56410.3 | 60657.3 | 0.121448 | 5.70737 | 9.84801 |
| R6 | 9091.69 | 1 | 78552.6 | 99665.6 | 0.058402 | 11.8685 | 14.9899 |
| Mean | 8270.958 | 1.5 | 65909.05 | 77144.38 | 0.094413 | 8.49662 | 12.46936 |
| SD | 1909.687 |  | 13899.2 | 14725.87 | 0.036906 | 3.615655 | 4.513338 |
| CV % | 23.08906 |  | 21.08846 | 19.08871 | 39.08991 | 42.55403 | 36.19542 |
