# Supplementary figures and images for: Ultra-performance hydrophilic interaction liquid chromatography coupled with tandem mass spectrometry for simultaneous determination of allopurinol, oxypurinol and lesinurad in rat plasma: Application to pharmacokinetic study in rats
Source: PLoS One. 2019 Mar 14;14(3):e0213786. doi: 10.1371/journal.pone.0213786 (PMC6417734; doi:10.1371/journal.pone.0213786)

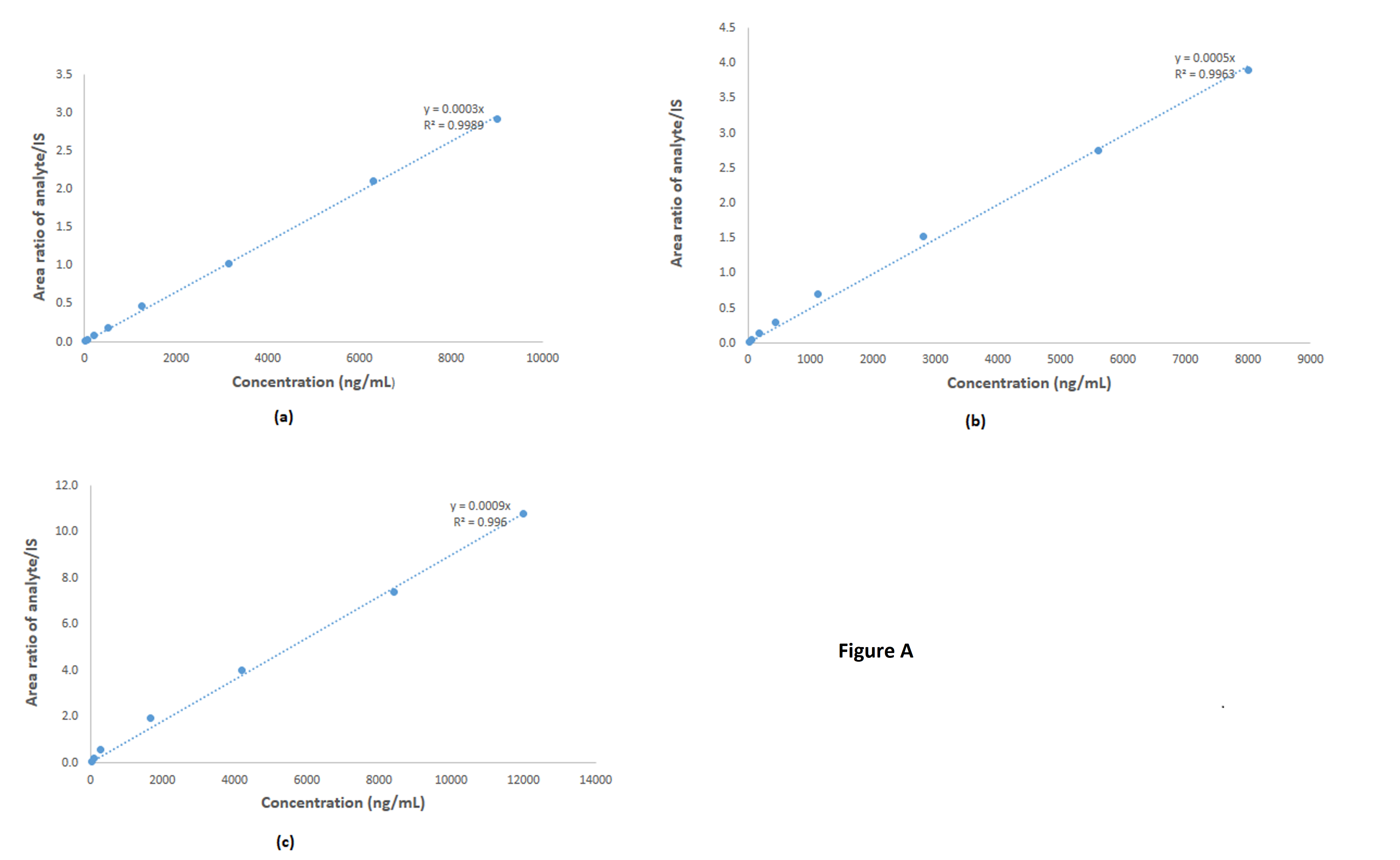

Supplement: S1 File — Stability data under different storage conditions (Table A). The individual animal pharmacokinetic data of ALP (Table B), OXP (Table C), and LES (Table D). (ZIP) [file pone.0213786.s001.zip › Figure A in S1 File.tif]
